# Evaluation of atherosclerosis as a risk factor in COPD patients by measuring the carotid intima-media thickness

**DOI:** 10.1186/s12947-023-00322-8

**Published:** 2024-01-10

**Authors:** Ali Firincioglulari, Hakan Erturk, Mujgan Firincioglulari, Cigdem Biber

**Affiliations:** 1 Department of Chest Diseases, Dr Burhan Nalbantoğlu State Hospital, Nicosia, Cyprus; 2 Department of Radiology, Health Sciences University, Ankara Atatürk Sanatoryum Training and Research Hospital, Ankara, Turkey; 3 Faculty of Dentistry, Department of Dentomaxillofacial Radiology, Cyprus International University, Nicosia, Cyprus; 4 Department of Chest Diseases, Health Sciences University, Ankara Atatürk Sanatoryum Training and Research Hospital, Ankara, Turkey

**Keywords:** COPD, Atherosclerosis, Stroke, CIMT, Carotid Bulbus

## Abstract

**Background:**

This study aimed to evaluate atherosclerosis as comorbidity by measuring the carotid (bulb and common carotid artery) Carotid intima-media thickness in COPD-diagnosed patients and to evaluate the relationship of atherosclerosis with the prevalence of COPD, hypoxemia and hypercapnia.

**Methods:**

This study was conducted out between January 2019-December 2019 consisting of a total of 140 participants (70 COPD-diagnosed patients-70 healthy individuals). The COPD-diagnosed patients have been planned according to the selection and diagnosis criteria as per the GOLD 2019 guide. It is planned to evaluate as per prospective matching case-control study of the carotid thickness, radial gas analysis, spirometric and demographic characteristics of COPD diagnosed patients and healthy individuals.

**Results:**

The average Carotid intima-media thickness in COPD patients was 0.8746±0.161 (*p*<0.05), and the thickness of the carotid bulb was 1.04±0.150 (*p*<0.05). In the control group, the average CCA intima-media thickness was 0.6650±0.139 (*p*<0.05), and the thickness of the carotid bulb was 0.8250±0.15(*p*<0.05) For the carotid thickness that has increased in COPD diagnosed patients a significant relationship is determined between hypoxemia (*p*<0.05) and hypercapnia(*p*<0.05). A significant relationship determined between CIMT and severity of COPD (*p*<0.05) The CIMT was high in COPD patients with hypoxemia and hypercapnia(*p*<0.05).

**Conclusion:**

Significant difference was determined between the severity (grades) of COPD (mild, moderate, severe, very severe) in carotid thickness. Also, CIMT was found to be high in patients who is in the early phases of the prevalence of COPD. In COPD-diagnosed patients, it was determined that severity of COPD, hypoxemia, hypercapnia and age were determining factors of atherosclerosis.

**Graphical Abstract:**

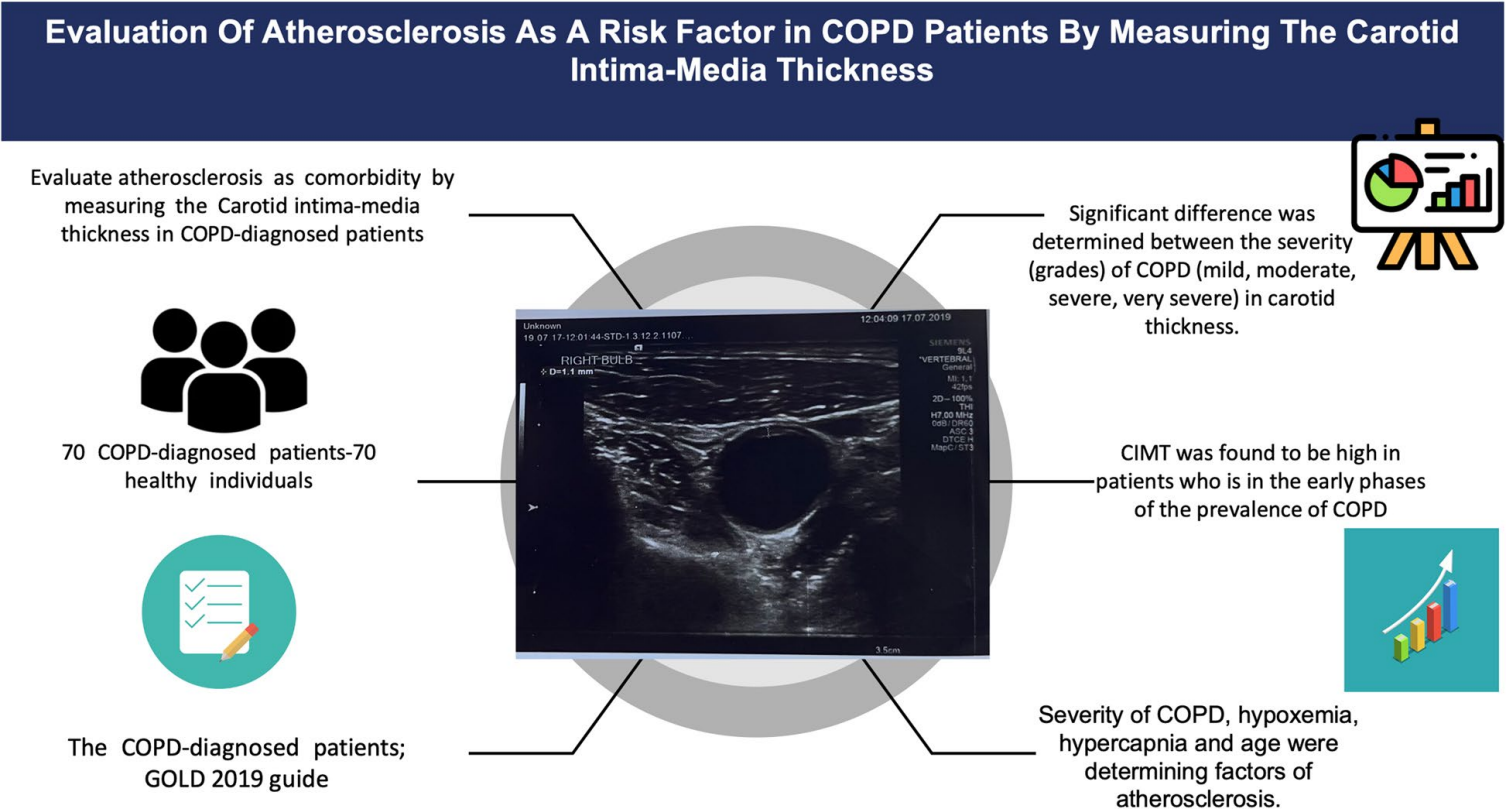

## Background

A common, preventable, and treatable condition known as chronic obstructive pulmonary disease (COPD) is described by recurrent respiratory symptoms and airflow restriction by cause of airway and/or alveolar abnormalities, which are typically brought on by prolonged exposure to harmful gases or particles [1]. COPD is often associated with comorbidities. Cardiovascular diseases are one of the most common comorbidities [2]. It has long been known that cardiovascular illness significantly increases the risk of morbidity and mortality in patients with COPD [3]. Moreover, COPD is a proven independent risk factor for atherosclerosis and cerebrovascular ischemic strokes caused by atherosclerosis [4].

Increased intima-media thickness, a sign of preclinical carotid atherosclerosis, indicates the presence of atherosclerosis and the risk of cardiovascular disease [5]. Carotid atherosclerosis indicates coronary artery diseases, peripheral artery diseases and cerebrovascular diseases is firmly correlated with coronary atherosclerosis [6, 7].

The structure by which increased carotid intima-media thickness is associated with COPD is not well known [8, 9]. COPD can be considered a disease that accelerates aging [10, 11]. The mechanism that accelerates aging and cellular aging play a role in the pathologic process of atherosclerosis [12]. The mechanism of aging in atherosclerosis and COPD has been verified by abbreviated length of leukocyte telomere [11–13]. In addition, low levels of systemic inflammation [9], excessive smoking frequency, inactive lifestyle, low FEV1 value (forced expiratory volume) [14], platelet activation, hypercoagulability and oxidative stresses contribute to atherosclerosis and aging [8, 15]. Chronic hypoxemia is related with atherosclerosis, increased risk of cardiovascular diseases and hyperlipidemia. The extent of atherosclerosis corresponds with the severity of hypoxia [16]. Abnormalities of extracellular matrix in the arterial wall cause elastin degradation and atherosclerosis [17]. Between risk factors, systemic inflammation is considered as the most prominent risk factor [14].

An increasing level of evidence has occurred that subclinical atherosclerotic lesions can be detected by ultrasonography which is the non-invasive imaging modality [18]. Carotid intima-media thickness can be measured with carotid Doppler ultrasound with effective, reproducible and variable methods [19]. Measurement of carotid intima-media thickness with ultrasound measures arterial wall thickening by dividing it into phases before luminal plug development [20]. The most accurate information on this subject is the measurement of the carotid bulb thickness. Because the first signs of atherosclerosis and thickening of the carotid intima-media first appear in carotid bulb [21, 22].

Increased carotid intima-media thickness is related with cardiovascular morbidity in COPD patients [5].

In the literature, there is no previous study conducted by measuring the carotid bulb thickness of atherosclerosis as a risk factor in patients with a diagnosis of COPD by doppler ultrasound.

Thus, this study aimed to evaluate atherosclerosis as comorbidity by measuring the carotid intima-media thickness in COPD patients and to determine the relationship between atherosclerosis and the prevalence (grade) of COPD, hypoxemia and hypercapnia.

For this purpose, carotid (intima-media of bulb and common carotid artery (CCA)) thickness, arterial blood gas analysis, spirometry characteristics and demographic characteristics of healthy individuals with COPD diagnosis and without COPD diagnosis who applied to Health Sciences University, Department of Chest Diseases, Atatürk Sanatoryum Training and Research Hospital were evaluated. The research was planned and carried out as a prospective case-control study.

## Methods

The study was approved Health Sciences University, Atatürk Sanatoryum Training and Research Hospital Ethical Review Board (IRB-/2012-KAEK-15/1885). All procedures applied in studies involving human participants were under the ethical standards of the institutional and/or national research committee and with the 1964 Helsinki declaration and its later amendments or comparable ethical standards.

140 participants [70 patients with a diagnosis of COPD (COPD diagnosed patients were selected from patients who were hospitalized in our hospital, whose discharge was planned, and who did not have an additional chronic disease) and 70 healthy individuals without a diagnosis of COPD (control)] who applied to the Health Sciences University, Department of Chest Diseases, Atatürk Sanatoryum Training and Research Hospital between January 2019 and December 2019 were included in the study as a prospective matched case-control study.

Chronic diseases other than COPD (chronic heart failure, hematological or oncological disease, chronic infection, coronary and peripheral artery disease), diabetes mellitus, pregnancy, liver and kidney failure, previous cerebrovascular disease, presence of diseases that cause platelet structure dysfunction, smoking, COPD patients whose clinical condition is unstable, patients with antihypertensive, antihyperlipidemic, diabetic drug use, as well as those with a history of chronic drug use were determined as the exclusion criteria of the study.

Pulmonary function tests and arterial blood gas values of patients with a diagnosis of COPD will be arterial blood gases and pulmonary function obtained during the hospitalization of the patients.

In the spirometry measurement, short-acting beta2 agonists, long-acting beta2 agonists and theophylline were not given to the patients 6-12 and 24 hours before the test. Before 6 hours until the test, exercises, heavy meals and smoking were not allowed. Lung volume after 1 second in forced expiration (FEV)1%, forced vital capacity (FVC%), FEV1/FVC, and Forced expiratory flow rate (FEF) 25-75% values were measured in pulmonary function tests. In spirometric measurements, the best value obtained at the end of at least 3 blows, as recommended by the European Respiratory Society, was considered [23]. The prevalence (grades) of COPD in the patients were determined as grades 1-2-3-4, namely (mild, moderate, severe, and very severe) based on FEV1% values according to GOLD 2019.

**Fig. 1:**
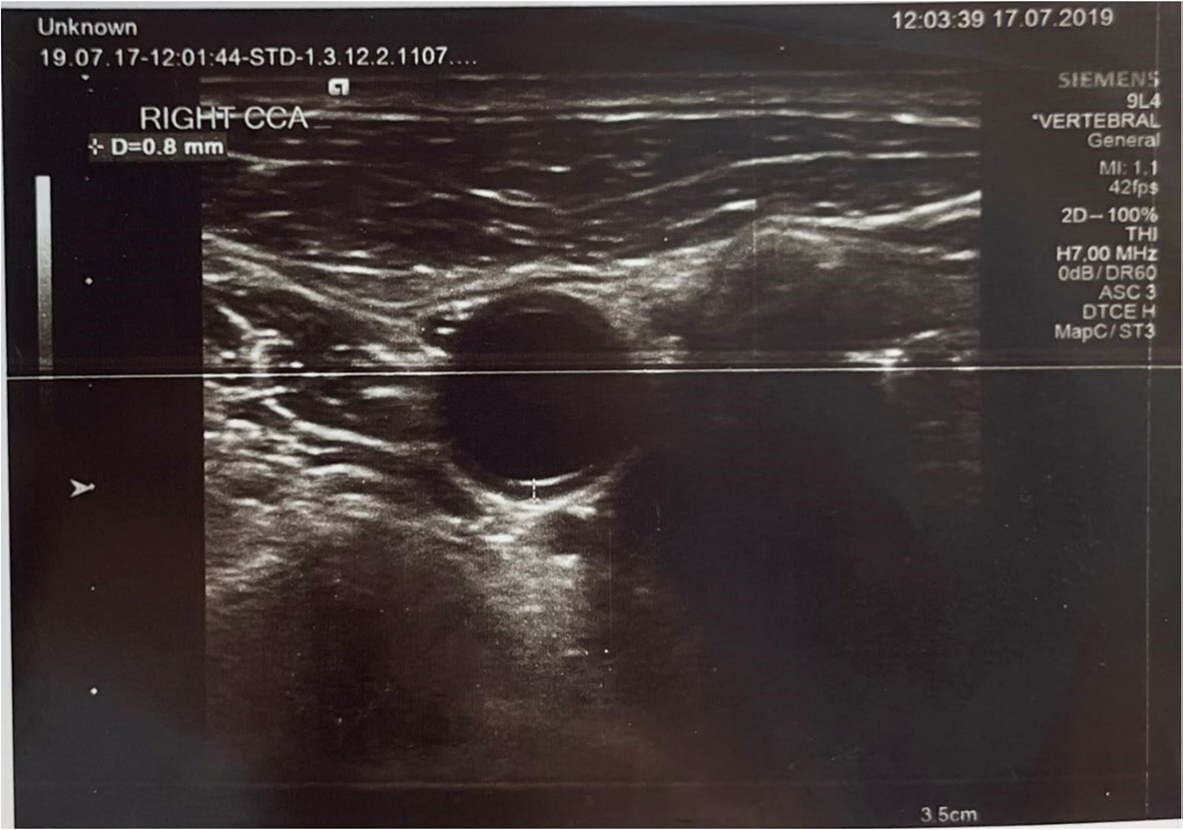
Measurement of right common carotid artery (CCA) in COPD diagnosed patient by ultrasound

**Fig. 2:**
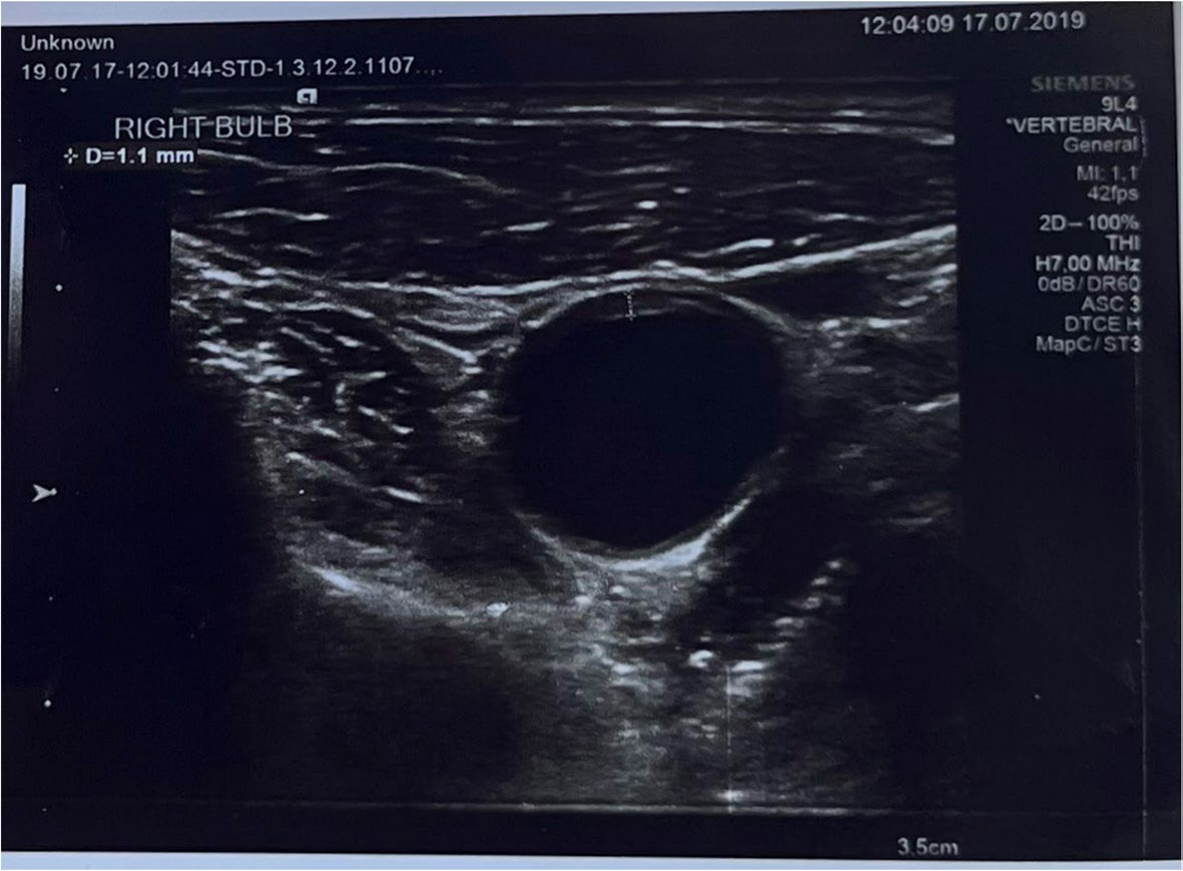
Measurement of right bulb of common carotid artery in COPD diagnosed patient by ultrasound

The thickness of the carotid intima-media was measured by ultrasound, taking the longitudinal and transverse axis, of both common carotid arteries and carotid bulbs in the supine position, and turning patients opposite side of the artery to be measured (Figs. 1 and 2). The average of the values measured from the right and left sides was taken. Measurements were made with Siemens HelixEvolution, using b-mode ultrasound, with a 10-12 Mhz linear transducer. When the thickness of the common carotid artery (CCA) intima media is greater than ≥0.8 mm, it is considered to be thickened, ie 'atherosclerosis' [24].

Arterial blood gases were taken after a rest period of at least 15 minutes in room air. The partial pressure of carbon dioxide (PaCO2) and partial pressure of oxygen (PaO2) and were evaluated in arterial blood gases.

### Statistical analysis

All statistical analyses were performed with SPSS for Windows version 22.0 (SPSS Inc., Chicago, IL). Pearson Chi-Square and Fischer's exact tests were used to evaluating categorical variables. The normal distribution of the data of numerical variables was evaluated with Shapiro Wilk, normality test and Q-Q charts. In the comparison of the groups, the Independent Sample T-test or one-way analysis of variance was used for normally distributed variables, and Mann-Whitney U or Kruskal-Wallis test was used for non-normally distributed variables. Risk factors were analyzed using a logistic regression model. The suitability of the logistic regression model was examined by the Hosmer-Lemeshow test. In the relationship between two continuous variables; Pearson correlation analysis was used for normally distributed variables and Spearman correlation analysis was used for non-normally distributed variables. The significance level was set at *p*<0.05.

## Results

The mean age was 61.6±8.68 years in patients with COPD and 52.55±10.9 years in the control group. Carotid intima-media thickened in 54 patients (77.15%) in the COPD group and 8 patients (11.5%) in the control group (*p*<0.001). In the COPD group, the mean CCA intima-media thickness was 0.8746±0.161 mm, and the carotid bulb thickness was 1.04±0.150 mm (*p*<0.05). In the control group, the mean common carotid artery intima-media thickness was 0.6650±0.139 mm and carotid bulb thickness was 0.8250±0.15 mm. (*p*<0.001). In the COPD group; While FEV1% was 47.45± 14.1%, FEV1/FVC was 57.04% ± 8.54, in the normal group; FEV1% was 87.12±13.73, FEV1/FVC was 81.74±5.31% (*p*<0.001). A statistically significant relationship was found between all these evaluation parameters (*p*<0.001). There was no significant difference only between the groups in BMI (*P*=0.174) (Table 1).

**Table 1:** Comparison of patients with and without COPD according to their personal characteristics, spirometric values and carotid intima media thickness. * shows statistical significance (*p* <0,05)

Measurements, Values, Markers	COPD group No=70	Non-COPD group No=70	Test-*p* value
Carotid intima-media thickness (CIMT).			
Normal	16 (22.85%)	62 (88.5%)	Chi square x2 (Fisher Exact)=60.82
Thickened (≥ 0.8mm)	54 (77.15%)	8 (11.5%)	*P*<0.001*
Carotid intima-media thickness (CCA) /mm			
Mean ± SD , Median	0.8736 mm ±0.161 (Mean)	0.6650 mm ±0.139 ( Mean)	Mann-Whitney U testi = 773.5
	0.9 mm(Median)	0.7 mm (Median)	*P*<0.001*
Carotid Bulb Thickness /mm	1.0464mm ±0.150 (Mean)	0.8250 mm ±0.156 (Mean)	Mann-Whitney U testi = 709
Mean ± SD, Median	1.05mm ( Median)	0.8 mm ( Median)	*P*<0.001*
Age/Year			
Mean ± SD	61.6 ± 8.68	52.55 ± 10.9 (Mean)	T-test= -5.475 *P*<0.001*
BMI:Kg/m2	26.33 ±5.06 ( Mean)	28.05± 5.54 (Mean)	Mann-Whitney U testi=2124
Mean ± SD, Median	26.17 (Median)	26.9 (Median)	*P*=0.174
Smoker	6 (8.57%)	47 (67.14%)	Chi square X2 ( Fisher Exact)=50.6
Non-smoker	64 (91.43%)	23 (32.86%)	*P*<.001*
Smoking ( Package/year)	36 ± 9.7 ( Mean)	20.9 ± 6.57 (Mean)	Mann Whitney U testi = 366.5
Mean ± SD,Median	35 ( Median)	20.0 (Median)	*P*<0.001
FEV1/FVC			
Mean ± SD, Median	57,04 ± 8.54 ( Mean)	81.74± 5.31 (Mean)	Mann-Whitney U testi =18.5
	58 ( Median)	82.0 (Median)	*P*<0.001
Fev1%			
Mean ± SD	47.45± 14.1 ( Mean)	87.12± 13.73 (Mean)	T-Test=16.8 *P*<0.001*
FVC%			
Mean ± SD	59.55± 13.95 ( Mean)	86.22± 11.65 (Mean)	T-Test=12.2 *P*<0.001*
FEF 25-75%			
Mean± SD Median	37.75± 16.82 (Mean)	83.84± 16.10 (Mean)	Mann Whitney U Testi=178
	36.5 ( Median)	87.0 (Median)	*P*<0.001*

*FEF25-75%* Forced expiratory flow rate 25%-75%, *FEV1* Lung volume after 1 second in forced expiration, *FVC* Forced vital capacity

In patients with a diagnosis of COPD with thickened Carotid intima-media (*n*=16); CCA CIMT 0.9370 mm ± 0.12, carotid bulb thickness 1.09 mm± 0.10 (*P*<0.001). In patients with COPD without CIMT thickening (*n*=54); CIMT thickness was 0.6594mm ± 0.75 and carotid bulb thickness was 0.8969 mm ± 0.17 (*P*<0.005). These values were statistically significant (*P*<0.001). A significant correlation was found between hypoxemia (*p*<0.05) and hypercapnia (p<0.05) in patients diagnosed with COPD with thickened carotid intima-media. A significant correlation was found between the two groups in FEV1% values (*p*<0.05). Cigarette smoking (package/year) was not statistically significant among COPD patients with thickened and normal Carotid intima-media (*P*=0.72). At the same time, smoking status was not statistically significant (*p*>0.05). There was no statistically significant difference between the groups in FEV1/FVC% values (*p*>0.05) (Table 2).

**Table 2:** Comparison of personal characteristics, spirometric values and carotid intima media diameter thickness degrees between groups with normal and thickened carotid intima media diameter in patients with COPD. * shows statistical significance (*p* <0,05)

Measurements, Values, Markers	COPD patients with normal CIMT (*n*=16)	Patients with a diagnosis of COPD with thickened Carotid intima-media (*n*=54)	Test-*p* value
Carotid Intima-Media Thickness (CCA) /mm	0.6594mm ± 0.75 (Mean)	0.9370 mm ± 0.12 (Mean)	Mann Whitney U Testi=0
Mean ± SD , Median	0.7mm (Median)	0.9mm (Median)	*p*<0.001*
Carotid Bulb Thickness /mm	0.8969 mm ± 0.17 (Mean)	1.09 mm± 0.10 (Mean)	Mann Whitney U Testi=137
Mean ± SD	0.9mm (Median)	1.1 mm (Median)	*p*<0.001*
Age/Year	56.87± 8.56 (Mean)	63.11± 8.27 (Mean)	T-Test=-2.62
Mean ± SD	57.00 (Median)	65.00 (Median)	*p*=0.01*
BMI:Kg/m2	27.36± 4.85 (Mean)	26.02± 5.12 (Mean)	T-Test=0.932
Mean ± SD	27.04 (Median)	25.91 (Median)	*p*=0.35
Non-smoker	1 (6.25%)	5 (9.25%)	Chi square x2 (Fisher Exact)=0.141
Smoker	15 (93.75)	49 (90.75%)	OR=0.65
			*p*=0.7
Smoking ( Package/year)	No=15	No=49	T-Test=-0.359
Mean ± SD	35.2 ± 8.69 (Mean)	36.24± 10.16 (Mean)	*p*=0.72
	35.00 (Median)	35.00 (Median)	
COPD duration/year	13.06 ± 3.94 (Mean)	15.70± 6.06 (Mean)	Mann Whitney U Testi=197.5
Mean± SD, Median	13.5 (Median)	15.00 (Median)	*p*=0.197
Hypoxemia:			Chi square x2 (Fisher Exact)=8.99
No	6 (37.5%)	4 (7.5%)	OR=7.5
Yes	10 (62.5%)	50 (92.5%)	*p*=0.003*
PaO2 mmHg	68.50 ± 12.7 (Mean)	57.53± 8.47 (Mean)	Mann Whitney U Testi=161
Mean ± SD, Median	64.6 (Median)	56.25 (Median)	*p*<0.001*
Hypercapnia :			Chi square x2 (Fisher Exact)=5.86
No	14 (87.5%)	29 (53.7%)	OR=6.0
Yes	2 (12.5%)	25 (46.3%)	*p*=0.015*
PaC02 mmHg	38.91 ± 4.80 (Mean)	44.39± 7.63 (Mean)	Mann Whitney U Testi=225
Mean ± SD, Median	35.70 (Median)	42.70 (Median)	*P*=0.04*
COPD Prevalence (degree)			
Mild-Moderate	9 (56.25%)	18 (33.4%)	Chi square x2 (Fisher Exact)=2.697
Severe-Very Severe	7 (43.75%)	36 (66.6%)	OR=2.5
Mean ± SD, Median			*p*=0.101
Fev1%	56.0 ± 9.4 (Mean)	44.92 ± 14.3 (Mean)	Mann Whitney U Testi=342
Mean ± SD, Median	57.5 (Median)	45.0 (Median)	*p*<0.001*
FEV1/FVC	56.87± 8.75 (Mean)	57.09± 8.56 (Mean)	T-Test=-0.89
Mean ± SD, Median	58.0 (Median)	58.00 (Median)	*p*=0.92
FVC%	66.81± 13.59 (Mean)	57.40± 13.43 (Mean)	T-Test=2.45
Mean ± SD	68.5 (Median)	55.5 (Median)	*p*=0.017*
FEF 25-75%	47.81 ± 19.27 (Mean)	34.77± 14.96 (Mean)	T-Test=2.86
Mean± SD	49.5 (Median)	35.5 (Median)	*p*=0.006*

CCA carotid intima-media thickness was significantly high in COPD patients with hypoxemia and hypercapnia(*p*<0.05). There was a statistically significant correlation between CCA and bulb carotid intima-media thicknesses in patients with mild-moderate and severe-very severe COPD prevalence (*p*<0.05) (Table 3).

**Table 3:** Comparison of carotid intima-media thickness in patients with COPD according to the prevalence of hypoxemia, hypercapnia and COPD. * shows statistical significance (*p* <0,05)

	CIMT Mean ± SD, Median	Carotid bulb thickness	Test-*p* value
Hypoxemia	0.72mm ± 0.14(Mean)	0.98mm± 0.21 (Mean)	CCA CIMT: Mann Whitney U Test=125
No (*N*= 10)	0.72mm (Median)	1.02mm (Median)	*p*=0.03*
Yes (*N*= 60)	0.898mm ± 0.15(Mean)	1.057mm± 0.13 (Mean)	Bulb CIMT: Mann Whitney U Test=249
	0.9mm (Median)	1.05mm (Median)	*p*=0.39
Hypercapnia			
No (*N*=43)	0.836mm ± 0.16 (Mean)	1.01mm± 0.157 (Mean)	CCA CIMT: Mann Whitney U Test=375
	0.85mm (Median)	1.00mm (Median)	*p*=0.012*
Yes (*N*=27)	0.93mm± 0.13 (Mean)	1.09mm± 0.12 (Mean)	Bulb CIMT: Mann Whitney U Test=358
	0.9mm (Median)	1.1mm ( Median)	*p*=0.007*
COPD Prevalence (degree)			
Mild-Moderate (*N*=27)	0.81mm± 0.16 (Mean)	1.005mm± 0.17 (Mean)	CCA CIMT: Mann Whitney U Test=394
	0.8mm (Median)	1.0mm (Median)	*p*=0.023*
Severe-Very Severe (*N*=43)	0.909mm±0.14 (Mean)	1.07mm± 0.12 (Mean)	Bulbus CIMT: Mann Whitney U Test=417
	0.9mm (Median)	1.1mm ( Median)	*p*=0.046*

In the logistic regression risk analysis in patients with COPD, the most important risk factors for CIMT were age (B=0.119) and Pao2 (B=-0.11) (*p*=0.022* and *p*= 0.019*). These values were found to be statistically significant. As a result, CIMT increased 1.126 times as age increased, and CIMT decreased 0.896 times as Pao2 increased. No significant risk was found for CIMT in other variables (Table 4).

**Table 4:** Multivariant logistic regression risk factors of carotid intima-media thickness in patients with COPD. * shows statistical significance (*p* <0,05)

	Beta coefficient (B)	Std. Error	Wald	Sig.	Exp(B)	95% Confidence Interval for Exp(B)	
						Lower Bound	Upper Bound
Intercept	3.711	6.334	0.343	0.558			
Age/Year	0.119	0.052	5.231	0.022*	1.126	1.017	1.246
PaO2 mmhg	-0.110	0.047	5.468	0.019*	0.896	0.817	0.982
PaCO2 mmhg	0.078	0.066	1.404	0.236	1.081	0.950	1.230
Fev1%	-0.061	0.038	2.551	0.110	0.941	0.873	1.014
Cigarette-package/year	-0.086	0.052	2.732	0.098	0.918	0.829	1.016

*PaO2* Partial pressure of oxygen, *PaCO2* Partial pressure of carbon dioxide

There was a significant negative correlation between PaO2 mm/hg and carotid thickening, CCA and bulb CIMT and FEV1% values in patients diagnosed with COPD, and a significant positive correlation between PaCO2mm/hg and Bulb thickness. All these values were statistically significant (*p*<0.05). There was no significant correlation between patients diagnosed with COPD with carotid thickening and cigarette packs/year(*p*>0.05) (Tables 5 and 6).

**Table 5:** Parametric pearson correlation

	Carotid thickening	Age	BMI	Cigarette-package/year	Fev1Fvc Ratio	Fvc Percentage	FEF 25-75 Percentage
Carotid thickening							
Pearson Correlation	1	.304	-0.112	0.046	0.011	-0.285	-0.328
Sig.(2-tailed)		0.11	0.355	0.720	0.929	0.017	0.006
N	70	70	70	64	70	70	70
Age							
Pearson Correlation	.304	1	-0.134	.0.085	-0.015	-0.054	-0.268
Sig.(2-tailed)	0.11		0.269	0.503	0.900	0.658	0.025
N	70	70	70	64	70	70	70
BMI							
Pearson Correlation	0.112	-0.134	1	-0.096	0.178	0.150	0.002
Sig.(2-tailed)	0.355	0.269		0.450	0.140	0.216	0.990
N	70	70	70	64	70	70	70
Cigarette-package/year							
Pearson Correlation	0.046	.0.085	-0.096	1	-0.287	-0.327	-0.357
Sig.(2-tailed)	0.720	0.503	0.450		0.021	0.008	0.004
N	64	64	64	64	64	64	64
Fev1Fvc Ratio							
Pearson Correlation	0.011	-0.015	0.178	-0.287	1	0.369	.0.458
Sig.(2-tailed	0.929	0.900	0.140	0.021		0.002	0.000
N	70	70	70	64	70	70	70
FVC Percentage							
Pearson Correlation	-0.285	-0.054	0.150	-0.327	0.369	1	0.545
Sig.(2-tailed)	0.017	0.658	0.216	0.008	0.002		0.000
N	70	70	70	64	70	70	70
FEF2575 Percentage							
Pearson Correlation	-0.328	-0.268	0.002	-0.357	.0.458	.0.545	1
Sig.(2-tailed)	0.006	0.025	0.990	0.004	0.000	0.000	
N	70	70	70	64	70	70	70

**Table 6:** Non-parametric spearman correlation

	Carotid thickening	CCA thickness	Bulb thickness	Fev1 percentage	COPD duration	Pao2 mmHg	PaCo2mmHg
Carotid thickening							
Pearson Correlation		0.737	0.501	-0.395	0.155	-0.456	0.349
Sig.(2-tailed)	1.000	0.000	0.000	0.001	0.199	0.000	0.003
N	70	70	70	70	70	70	70
CCA thickness							
Pearson Correlation	0.737	1.000	0.747	-0.420	0.276	-0.445	0.396
Sig.(2-tailed)	0.000		0.000	0.000	0.021	0.000	0.001
N	70	70	70	70	70	70	70
Bulb thickness							
Pearson Correlation	0.501	0.747	1.000	-0.337	0.251	-0.278	0.424
Sig.(2-tailed)	0.000	0.000		0.004	0.036	0.020	0.000
N	70	70	70	70	70	70	70
Fev1 percentage							
Pearson Correlation	-0.395	-0.420	-0.337	1.000	-0.231	0.424	-0.458
Sig.(2-tailed)	0.001	0.000	0.004		0.054	0.000	0.000
N	70	70	70	70	70	70	70
COPD duration							
Pearson Correlation	0.155	0.276	0.251	-0.231	1.000	-0.072	0.116
Sig.(2-tailed)	0.199	0.021	0.036	0.054		0.552	0.340
N	70	70	70	70	70	70	70
Pao2 mmHg							
Pearson Correlation	-0.456	-0.445	-0.278	0.424	-0.072	1.000	-0.443
Sig.(2-tailed)	0.000	0.000	0.000	0.000	0.552		0.000
N	70	70	70	70	70	70	70
PaCo2mmHg							
Pearson Correlation	0.349	0.396	0.424	-0.458	0.116	-0.443	1.000
Sig.(2-tailed)	0.003	0.001	0.000	0.000	0.340	0.000	
N	70	70	70	70	70	70	70

## Discussion

COPD is a preventable and treatable disease characterized by an inflammatory response in the lungs and airways to harmful gases and particles, persistent and often progressive airflow limitation, exacerbations, and comorbidities [1]. COPD is considered a systemic disease with extrapulmonary effects. COPD arises from the interaction between genetic susceptibility and environmental factors [25]. Comorbidities play an important role in COPD and contribute significantly to morbidity and mortality [26]. Cardiovascularly, one or more comorbidities such as angina, cerebrovascular stroke, arrhythmia, ventricular hypertrophy, and myocardial infarction may appear, and these comorbidities greatly reduce the survival of COPD patients [27]. Studies are showing that there is an increased risk of cardiovascular disease in COPD patients and therefore the risk of cardiovascular death in COPD is high [28, 29]. Studies have reported that up to 40% of deaths in COPD patients are due to cardiovascular diseases [30, 31]. Patients with mild COPD have a higher ratio of dying from cardiovascular diseases rather than respiratory failure [32]. There is a significant overlap between the risk factors associated with the development of COPD and atherosclerotic vascular disease. Clinically, there is a strong correlation between FEV1 and cardiovascular morbidity and mortality. However, COPD patients have a high risk of fatal myocardial infarction regardless of smoking status [33]. A large population study has shown that patients with severe to very severe COPD have a >2 times higher risk of cardiovascular disease, a 1.6 times higher prevalence of hypertension, and a higher risk of hospitalization [34]. It has been reported that the presence of persistent low-grade systemic inflammation found in COPD and atherosclerotic cardiovascular diseases may be the factor driving both pathologies [8, 14, 28]. The increased serum leukocyte, platelet and fibrinogen levels and the presence of increased cytokines, chemokines and acute phase proteins in COPD indicate that COPD is characterized by persistent systemic inflammation not only in the lungs [35, 36]. It has been reported that low-grade systemic inflammation is present even in non-smokers with COPD [37].

Carotid intima-media thickness has an important place in determining atherosclerosis. Carotid intima-media thickness measurement, values that differ slightly from each other have been determined in many studies. In these studies, it is recommended to average the measurements made from the right and left main carotid artery posterior wall for each patient and to evaluate them by comparing them with the values obtained from normal subjects [38, 39].

A consensus has been reached for CCA CIMT. In a meta-analysis [39], CIMT was found to be 0.76 mm ± 0.17 more in patients with myocardial infarction, 0.77 mm ± 0.18 more in those with a cerebrovascular stroke, and more than 0.82 mm ± 0.17 in those with both myocardial infarction and cerebrovascular stroke. In the meta-analysis of Lorenz et al. [40], the risk of myocardial infarction increases from 10% to 15%, and the risk of the ischemic cerebrovascular event increases from 13% to 18% with each 0.1 mm increase in IMT.

The 'bulb’ region where the CCA expands and bifurcates is important in the measurement of atherosclerosis.^40^ Because the blood flow in the bulb region is the slowest compared to other regions, it also has turbulent flow [41–43]. Therefore, atherosclerosis and carotid artery plaques first appear in the carotid bulb region [40]. However, since the bulb thickness is difficult to measure and experienced hands are required, the studies are fewer. The mean carotid bulb thickness was found to be 0.8925 ± 0.539 mm for the occurrence of cerebrovascular disease in the analyzes made with computer methods and programs through the data obtained from the patients [44]. No study in the literature evaluated atherosclerosis by measuring the carotid bulb thickness in patients with COPD.

In the literature, there are studies stating that coronary artery diseases are less common in COPD, as well as studies reporting that increased CIMT is observed in COPD patients and cardiovascular diseases are more common [45–47]. Eickoff et al. [48] measured higher CIMT in stable COPD patients than in healthy smokers or nonsmokers. Kim et al. [49] also found CIMT to be significantly higher in newly diagnosed and untreated COPD patients compared to healthy individuals. Similarly, van Gestel et al. [18] reported that COPD was associated with increased CIMT in patients undergoing vascular surgery, and increased CIMT was associated with increased cardiovascular mortality. In this study, in accordance with the literature, significantly increased CIMT and Carotid bulb thickness were observed in patients with COPD compared to healthy individuals (*p*< 0.001).

The risk of death due to myocardial infarction is significantly higher in patients with COPD, and it has been reported to be more pronounced regardless of age, gender, and smoking history [50]. In addition, Pobeha et al. reported that there was no difference between the CIMTs of COPD patients at different stages and that atherosclerosis observed in COPD started in the early period. Accordingly, they recommended that patients be evaluated in terms of concomitant atherosclerosis in the early period of COPD [9]. It has been reported in the literature that there is a strong correlation between clinically impaired lung function (FEV1%) and cardiovascular morbidity and mortality [14, 28, 49, 50]. Eickhoff et al. [49] found an inverse correlation between the increase in CIMT and FEV1% in patients with COPD. Recent studies have reported a change in FEV1% and its association with vascular wall stiffness and atherosclerosis in smokers with airflow limitation [50, 51]. Zureik et al. [52] reported that the disorder in FEV1% is associated with hardening of the vessel wall, endothelial dysfunction and atherosclerosis. Sin et al. [50] stated that the risk of cardiovascular death was higher in patients with low FEV1%. Also in our study, a negative correlation was observed between FEV1% and CIMT increase.

However, it has been reported that increased cholesterol and fasting blood sugar levels, which are risk factors for atherosclerosis, do not increase in COPD patients compared to healthy individuals [46, 53, 54]. Basili et al. found lipid levels to be significant in patients with COPD compared to healthy individuals [55]. The relationship between Diabetes Mellitus and COPD is also not clear [8]. In previous studies, it has been reported that total cholesterol, triglyceride and LDL cholesterol levels other than HDL are significantly lower in patients with COPD than in healthy individuals [47, 56, 57]. It is thought that the decrease in serum lipid levels observed in patients with COPD may be caused by anaerobic changes in carbohydrate and lipid metabolism after the hypoxic process [55].

Our study results support the knowledge that inflammation in COPD is more effective than the effect of smoking on the intima-media thickness of the carotid as stated in the study of Eickhoff et al. [49] in 60 patients with stable COPD and 20 healthy smokers and 20 healthy non-smokers. Smoking (package/year) among COPD patients in our study was not statistically significant in terms of CIMT (*p*>0.05).

In the study of Hafez et al. [58], patients with COPD and healthy individuals were compared and CIMT was discussed. Spirometric values and demographic characteristics of the patients and control group were examined. As in our study, the most important risk factors for CIMT in the Logistic regression risk analysis in patients with COPD were age and PaO2.

In this study, a significant correlation was found between hypoxemia (*p*<0.05) and hypercapnia (*p*<0.05) in patients diagnosed with COPD with carotid thickening. These results may lead to atherosclerosis by increasing the systemic inflammation of hypoxemia, oxidative stress, increased foam cell production and upregulation of cell adhesion molecules [16, 59, 60]. Similar results were reported by Ozbay et al. [61] According to their study, oxygen saturations decreased in patients with a diagnosis of COPD with thickened Carotid intima-media [62].

In studies conducted on patients with a diagnosis of COPD with carotid thickening, the distribution of COPD grades (mild, moderate, severe and very severe) was not found to be significant in determining carotid thickness [9, 62]. In our study, a significant correlation was found between CIMT and COPD prevalence (degree) in patients diagnosed with COPD with carotid thickening (*p*<0.05). However, considering the carotid thicknesses measured in mild and moderate COPD groups in our study, the mean carotid CCA thickness was 0.81mm±0.16, and the carotid bulb thickness was 1.005mm±0.17. These values were statistically significant (*p*<0.05). These values can be considered as an indication of the onset of carotid thickness, even in the early stages of COPD (grade 1-2).

In a meta-analysis [39], CIMT was found to be 0.76 mm ± 0.17 in patients with myocardial infarction, 0.77 mm ± 0.18 in those with a cerebrovascular stroke, and more than 0.82 mm ± 0.17 in those with both myocardial infarction and cerebrovascular stroke. In the study of Hafez et al. [58], CIMTs were 0.63±0.076 mm in the control group and 0.85±0.18 mm in the COPD group. In our study, the mean CCA intima-media thickness in the COPD group was 0.8746±0.161, and the mean CCA intima-media thickness in the control group was 0.6650±0.139. In other studies, CIMTs give similar results in COPD patients [5, 18, 49, 61].

In this study, carotid bulb thickness was 1.04±0.150 (*p*<0.05) in the COPD group and 0.8250±0.15 in the control group (*p*<0.05). Carotid bulb thickness was 1.09 mm± 0.10 (*P*<0.05) in COPD patients with Carotid intima-media thickened, and 0.8969 mm ± 0.17 in patients with COPD without CIMT thickening (*P*<0.05). There was a significant negative correlation between bulb CIMT and FEV1% values ​​and Pao2 mm/hg, and a significant positive correlation between PaCO2 mm/hg and COPD duration. Bulb thicknesses were found to be higher than CCA CIMT in our study. The increase in these values ​​should be considered a normal increase, because the blood flow in the bulb region is the slowest compared to other regions of the carotid, and it also has turbulent flow [41–43]. Therefore, atherosclerosis and carotid artery plaques first appear in the bulb region of the carotid [40]. In COPD patients with hypoxemia, carotid bulb intima-media thickness was not significantly higher than the group without hypoxemia (*p*>0.05). Because the carotid bulb thickness was found to be higher in the group without hypoxemia (0.98mm±0.21). This may be attributed to the first occurrence of atherosclerosis in the carotid bulb region [41–43].

As the limitations of the study, the number of female patients was low in patients with COPD. Patients with a diagnosis of COPD are less common in the community. In the patient group diagnosed with COPD, generally, male patients were included in the study. Another limitation is that although the group with COPD without additional diseases and the control group were selected, lipid profiles, which are independent risk factors for atherosclerosis, and 3-month blood glucose follow-ups could not be evaluated. However, it has been reported that increased cholesterol and fasting blood sugar levels, which are risk factors for atherosclerosis, do not increase in COPD patients compared to healthy individuals [46, 55–57].

In addition, contrast ultrasound has already been widely adopted not only to define myocardial borders and also perfusion [63, 64]. So it can be thought that contrast ultrasound might help further to stratify IMT characteristics. More studies with the use of contrast ultrasound would be useful.

## Conclusion

According to this study, a significant difference was found between the severity (grades) of COPD (mild, moderate, severe, very severe) in carotid thickness. Also, CIMT was found to be high even in patients in the early stages (degree) of the prevalence of COPD. It was determined that FEV1% values decreased as the carotid thickness increased. Age, hypoxemia, hypercapnia and the prevalence (degree) of COPD were determined as atherosclerosis predictors in COPD patients. Smoking status was not seen as a determining factor.

In the literature, there is no study conducted by measuring the carotid bulb thickness of atherosclerosis as a risk factor in patients with a diagnosis of COPD by Doppler ultrasound. It is recommended that patients diagnosed with COPD be screened for atherosclerosis at an early stage from the onset of the disease. In addition, interventional studies targeting atherosclerosis in COPD patients and studies to evaluate the effects of early oxygen therapy on carotid thickness are needed.

## Data Availability

The datasets used and/or analysed during the current study are available from the corresponding author on reasonable request.

## Declarations

Ethics approval and consent to participate

The study was approved Health Sciences University, Atatürk Sanatoryum Training and Research Hospital Ethical Review Board (IRB-/2012-KAEK-15/1885). All procedures applied in studies involving human participants were under the ethical standards of the institutional and/or national research committee and with the 1964 Helsinki declaration and its later amendments or comparable ethical standards.
